# Supplementary material for: Identification of a carbohydrate recognition motif of purinergic receptors
Source: eLife. 2023 Nov 13;12:e85449. doi: 10.7554/eLife.85449 (PMC10642967; doi:10.7554/eLife.85449)
Supplement: Figure 1—source data 1. [file elife-85449-fig1-data1.docx]

Note: EC50s are measured in the calcium mobilization assay. Number of data points, agonist used and statistical significance are detailed, ns not significant.

**Figure 1*—*source data 1.** Potency of UDP or UDP-Glc in HEK293 cells expressing P2Y14

| **Construct** | **Agonist** | **EC50 (nM)** | ***n*** | **Statistics** |
| --- | --- | --- | --- | --- |
| P2Y14 | UDP | 50.9 ± 6.1 | 5 | T.TEST |
|  | UDP-Glc | 40.3 ± 1.5 | 12 | ns |
