# Supplementary material for: Identification of a carbohydrate recognition motif of purinergic receptors
Source: eLife. 2023 Nov 13;12:e85449. doi: 10.7554/eLife.85449 (PMC10642967; doi:10.7554/eLife.85449)
Supplement: Figure 1—source data 2. [file elife-85449-fig1-data2.docx]

Note: EC50s are measured in the calcium mobilization assay. Number of data points, agonist used and statistical significance are detailed, ns not significant.

**Figure 1*—*source data 2.** Potency of UDP or UDP-Glc in HEK293 cells expressing P2Y14 WT and mutants.

| **Agonist** | **Construct** | **EC50 (nM)** | ***n*** | **Statistics** | **Comment** |
| --- | --- | --- | --- | --- | --- |
| UDP | P2Y14-WT | 50.9 ± 6.1 | 5 | T.TEST |  |
|  | P2Y14-K77A | 819.9 ± 59.9 | 3 | *P* < 0.0001 | WT vs. K77A |
|  | P2Y14-D81A | 59.9 ± 6.1 | 3 | ns | WT vs. D81A |
|  | P2Y14-N90A | 40.6 ± 2.3 | 3 | ns | WT vs. N90A |
|  | P2Y14-I170A | 70.7 ± 9.6 | 3 | ns | WT vs. I170A |
|  | P2Y14-E278A | 42.2 ± 2.5 | 3 | ns | WT vs. E278A |
| UDP-Glc | P2Y14-WT | 40.3 ± 1.5 | 12 | T.TEST |  |
|  | P2Y14-K77A | 1930.0± 348.7 | 8 | *P* < 0.0001 | WT vs. K77A |
|  | P2Y14-D81A | 671.9 ± 44.4 | 8 | *P* < 0.0001 | WT vs. D81A |
|  | P2Y14-N90A | 51.2 ± 6.6 | 4 | *P* < 0.05 | WT vs. N90A |
|  | P2Y14-I170A | 48.8 ± 6.0 | 3 | ns | WT vs. I170A |
|  | P2Y14-E278A | 60.2 ± 3.6 | 8 | *P* < 0.0001 | WT vs. E278A |
