# Supplementary material for: Identification of a carbohydrate recognition motif of purinergic receptors
Source: eLife. 2023 Nov 13;12:e85449. doi: 10.7554/eLife.85449 (PMC10642967; doi:10.7554/eLife.85449)
Supplement: Figure 1—source data 3. [file elife-85449-fig1-data3.docx]

Note: EC50s are measured in the calcium mobilization assay. Number of data points, agonist used and statistical significance are detailed, ns not significant.

**Figure 1*—*source data 3.** Comparation of EC50s for UDP-Glc and UDP in HEK293 cells expressing P2Y14 mutants.

| **Construct** | **Agonist** | **EC50**  **(Fold of WT)** | ***n*** | **Statistics** | **Comment** |
| --- | --- | --- | --- | --- | --- |
| P2Y14-K77A | UDP | 14.3 ± 2.5 | 3 | T.TEST |  |
|  | UDP-Glc | 64.1 ± 9.9 | 4 | *P* < 0. 01 | UDP vs. UDP-Glc |
| P2Y14-D81A | UDP | 1.0 ± 0.0 | 3 |  |  |
|  | UDP-Glc | 18.3 ± 1.7 | 4 | *P* < 0. 001 | UDP vs. UDP-Glc |
| P2Y14-E278A | UDP | 0.7 ± 0.1 | 3 |  |  |
|  | UDP-Glc | 1.6 ± 0.1 | 4 | *P* < 0.01 | UDP vs. UDP-Glc |
