# Supplementary material for: Identification of a carbohydrate recognition motif of purinergic receptors
Source: eLife. 2023 Nov 13;12:e85449. doi: 10.7554/eLife.85449 (PMC10642967; doi:10.7554/eLife.85449)
Supplement: Figure 2—source data 1. [file elife-85449-fig2-data1.docx]

Note: EC50s are measured in the calcium mobilization assay. Number of data points, agonist used and statistical significance are detailed, ns not significant.

**Figure 2*—*source data 1.** Potency of UDP-Glc in HEK293 expressing P2Y14 WT and mutants.

| **Agonist** | **Construct** | **EC50 (nM)** | ***n*** | **Statistics** | **Comment** |
| --- | --- | --- | --- | --- | --- |
| UDP-Glc | P2Y14-WT | 40.3 ± 1.5 | 12 | T.TEST |  |
|  | P2Y14-K77A | 1930.0 ± 348.7 | 8 | *P* < 0.0001 | WT vs. K77A |
|  | P2Y14-D81A | 671.9 ± 44.4 | 8 | *P* < 0.0001 | WT vs. D81A |
|  | P2Y14-R253A | 808.6 ± 43.6 | 4 | *P* < 0.0001 | WT vs. R253A |
|  | P2Y14-T257A | 504.9 ±15.9 | 4 | *P* < 0.0001 | WT vs. T257A |
|  | P2Y14-K277A | 734.8 ± 12.8 | 8 | *P* < 0.0001 | WT vs. K277A |
|  | P2Y14-E278A | 60.2 ± 3.6 | 8 | *P* < 0.0001 | WT vs. E278A |
