# Supplementary material for: Identification of a carbohydrate recognition motif of purinergic receptors
Source: eLife. 2023 Nov 13;12:e85449. doi: 10.7554/eLife.85449 (PMC10642967; doi:10.7554/eLife.85449)
Supplement: Figure 3—source data 1. [file elife-85449-fig3-data1.docx]

Note: EC50s are measured in the calcium mobilization assay. Number of data points, agonist used and statistical significance are detailed, ns not significant.

**Figure 3-source data 1.** Potency of UDP-GlcA, UDP-GlcNAc and UDP-Gal in HEK293 expressing P2Y14.

| **Construct** | **Agonist** | **EC50(nM)** | ***n*** | **Statistics** | **Comment** |
| --- | --- | --- | --- | --- | --- |
| P2Y14 | UDP-Glc | 40.3 ± 1.5 | 12 | T.TEST |  |
| P2Y14 | UDP-Gal | 78.3 ± 9.2 | 4 | *P* < 0.0001 | UDP-Glc vs. UDP-Gal |
| P2Y14 | UDP-GlcA | 59.9 ± 4.8 | 6 | *P* < 0. 001 | UDP-Glc vs. UDP-GlcA |
| P2Y14 | UDP-GlcNAc | 184.4 ± 11.8 | 3 | *P* < 0.0001 | UDP-Glc vs. UDP-GlcNAc |
