# Supplementary material for: Identification of a carbohydrate recognition motif of purinergic receptors
Source: eLife. 2023 Nov 13;12:e85449. doi: 10.7554/eLife.85449 (PMC10642967; doi:10.7554/eLife.85449)
Supplement: Figure 3—source data 2. [file elife-85449-fig3-data2.docx]

Note: EC50s are measured in the calcium mobilization assay. Number of data points, agonist used and statistical significance are detailed, ns not significant.

**Figure 3*—*source data 2.** Potency of UDP-GlcA, UDP-GlcNAc and UDP-Gal in HEK293 expressing P2Y14 WT and mutants.

| **Agonist** | **Construct** | **EC50 (nM)** | ***n*** | **Statistics** | **Comment** |
| --- | --- | --- | --- | --- | --- |
| UDP-GlcA | P2Y14-WT | 59.9 ± 4.8 | 6 | T.TEST |  |
|  | P2Y14-K77A | > 3000 | 4 |  |  |
|  | P2Y14-D81A | 171.7 ± 9.8 | 4 | *P* < 0.0001 | WT vs. D81A |
|  | P2Y14-K277A | > 3000 | 4 |  |  |
|  | P2Y14-E278A | 38.2 ± 2.2 | 4 | *P* < 0. 01 | WT vs. E278A |
| UDP-GlcNAc | P2Y14-WT | 184.4 ± 11.8 | 3 | T.TEST |  |
|  | P2Y14-K77A | > 3000 | 3 |  |  |
|  | P2Y14-D81A | 2997 ±173.9 | 3 | *P* < 0.0001 | WT vs. D81A |
|  | P2Y14-K277A | 548.7 ± 74.4 | 4 | *P* < 0. 01 | WT vs. K277A |
|  | P2Y14-E278A | 624.2 ± 101.6 | 4 | *P* < 0. 05 | WT vs. E278A |
| UDP-Gal | P2Y14-WT | 78.3 ± 9.2 | 4 | T.TEST |  |
|  | P2Y14-K77A | 1964.0 ± 46.9 | 4 | *P* < 0.0001 | WT vs. K77A |
|  | P2Y14-D81A | 824.1 ± 64.3 | 4 | *P* < 0.0001 | WT vs. D81A |
|  | P2Y14-K277A | 2261.0 ± 347.6 | 4 | *P* < 0.001 | WT vs. K277A |
|  | P2Y14-E278A | 99.4 ± 11.6 | 4 | ns | WT vs. E278A |
