# Supplementary material for: Identification of a carbohydrate recognition motif of purinergic receptors
Source: eLife. 2023 Nov 13;12:e85449. doi: 10.7554/eLife.85449 (PMC10642967; doi:10.7554/eLife.85449)
Supplement: Figure 4—source data 1. [file elife-85449-fig4-data1.docx]

Note: EC50s are measured in the calcium mobilization assay. Number of data points, agonist used and statistical significance are detailed, ns not significant.

**Figure 4*—*source data 1.** ADP-Glc, ADP-GlcA and ADP-Man in HEK293 expressing P2Y12 WT and mutants.

| **Agonist** | **Construct** | **EC50 (μM)** | ***n*** | **Statistics** | **Comment** |
| --- | --- | --- | --- | --- | --- |
| ADP-Glc | P2Y12-WT | 3.4 ± 0.4 | 4 | T.TEST |  |
|  | P2Y12-K80A | 96.9 ± 14.4 | 4 | *P* < 0.001 | WT vs. K80A |
|  | P2Y12-D84A | 29.3 ± 1.2 | 4 | *P* < 0.0001 | WT vs. D84A |
|  | P2Y12-F277A | 15.4 ± 1.1 | 4 | P < 0.0001 | WT vs. F277A |
|  | P2Y12-K280A | 41.8 ± 3.0 | 4 | *P* < 0.0001 | WT vs. K280A |
|  | P2Y12-E281A | 75.3 ± 10.1 | 4 | *P* < 0.01 | WT vs. E281A |
| ADP-GlcA | P2Y12-WT | 1.3 ± 0.1 | 3 | T.TEST |  |
|  | P2Y12-K80A | 35.2 ± 0.5 | 3 | P < 0.0001 | WT vs. K80A |
|  | P2Y12-D84A | 9.4 ± 0.3 | 3 | P < 0.0001 | WT vs. D84A |
|  | P2Y12-F277A | 5.0 ± 0.4 | 3 | *P* < 0.01 | WT vs. F277A |
|  | P2Y12-K280A | 18.1 ± 3.3 | 3 | P < 0.01 | WT vs. K280A |
|  | P2Y12-E281A | 28.2 ± 4.1 | 3 | P < 0.01 | WT vs. E281A |
| ADP-Man | P2Y12-WT | 12.3 ± 0.9 | 4 |  |  |
|  | P2Y12-K80A | > 150 | 4 |  |  |
|  | P2Y12-D84A | > 150 | 4 |  |  |
|  | P2Y12-F277A | 128.1 ± 17.6 | 3 | *P* < 0.001 | WT vs. F277A |
|  | P2Y12-K280A | > 150 | 4 |  |  |
|  | P2Y12-E281A | > 150 | 4 |  |  |
