# Supplementary figures and images for: Identification of a carbohydrate recognition motif of purinergic receptors
Source: eLife. 2023 Nov 13;12:e85449. doi: 10.7554/eLife.85449 (PMC10642967; doi:10.7554/eLife.85449)

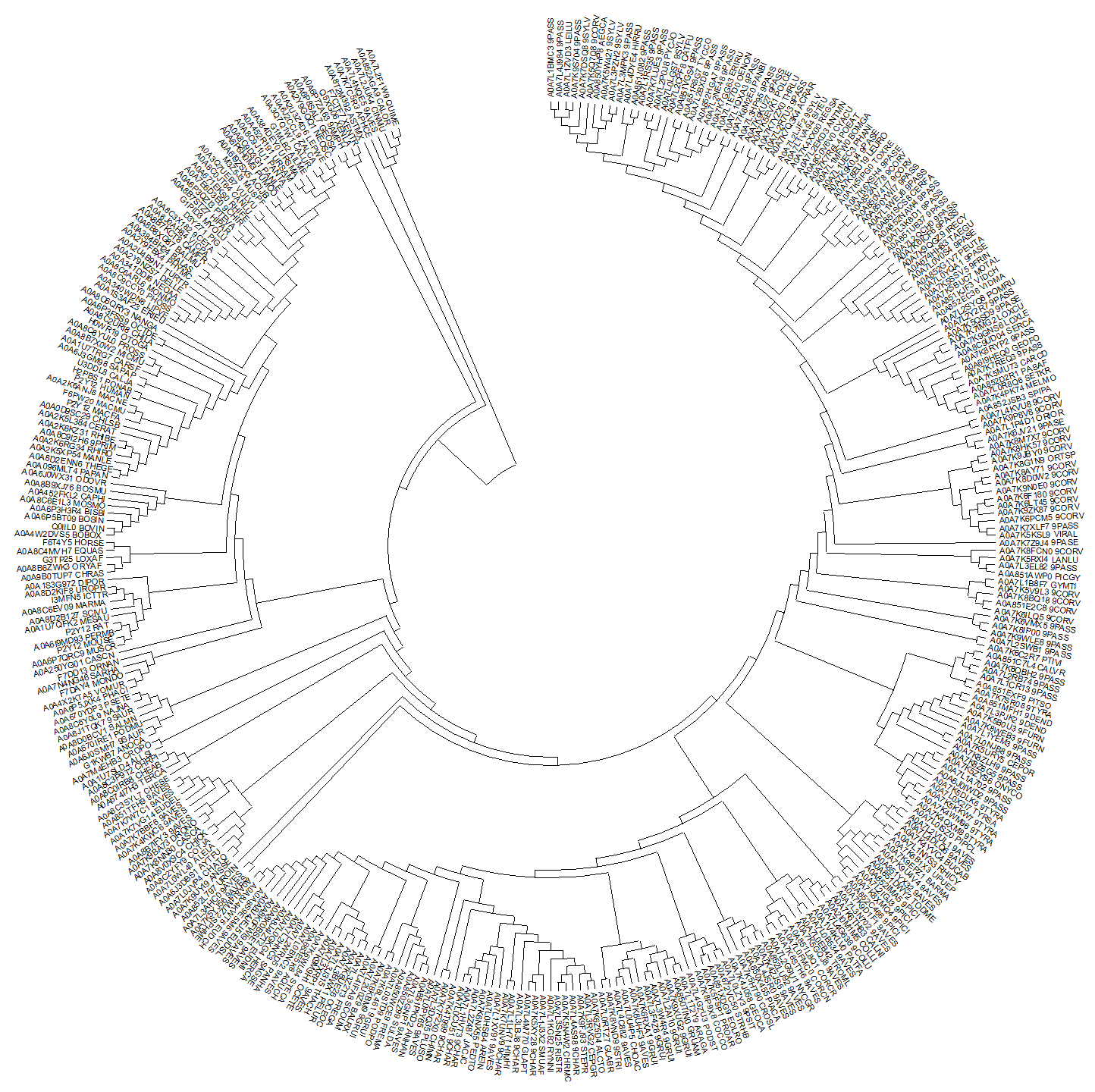


**Figure 4*—*source data 2.** The neighbor joining tree of P2Y12 showing evolutionary range of species.

Supplement: Figure 4—source data 2. [file elife-85449-fig4-data2.docx]

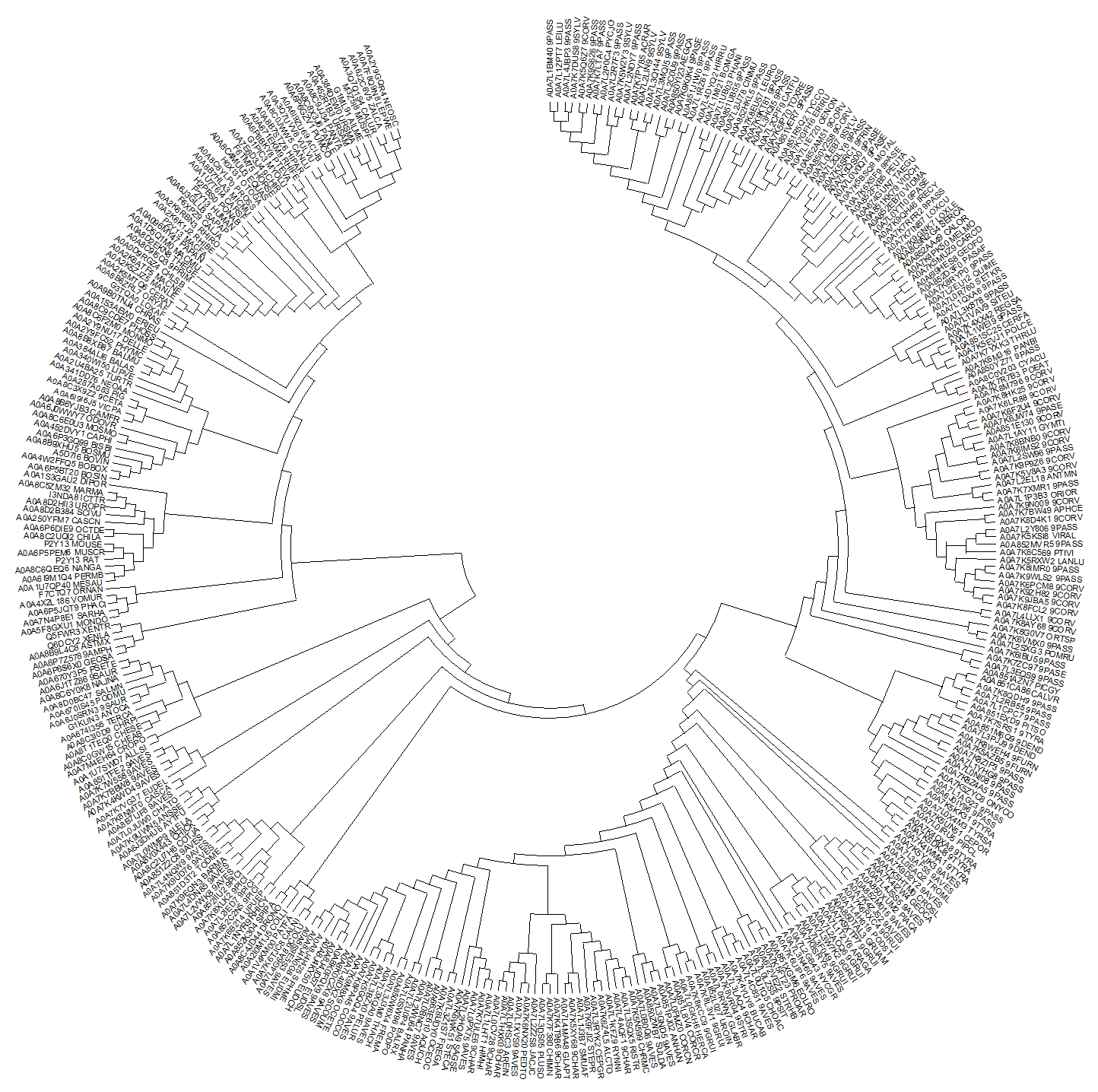


**Figure 4*—*source data 3.** The neighbor joining tree of P2Y13 showing evolutionary range of species.

Supplement: Figure 4—source data 3. [file elife-85449-fig4-data3.docx]

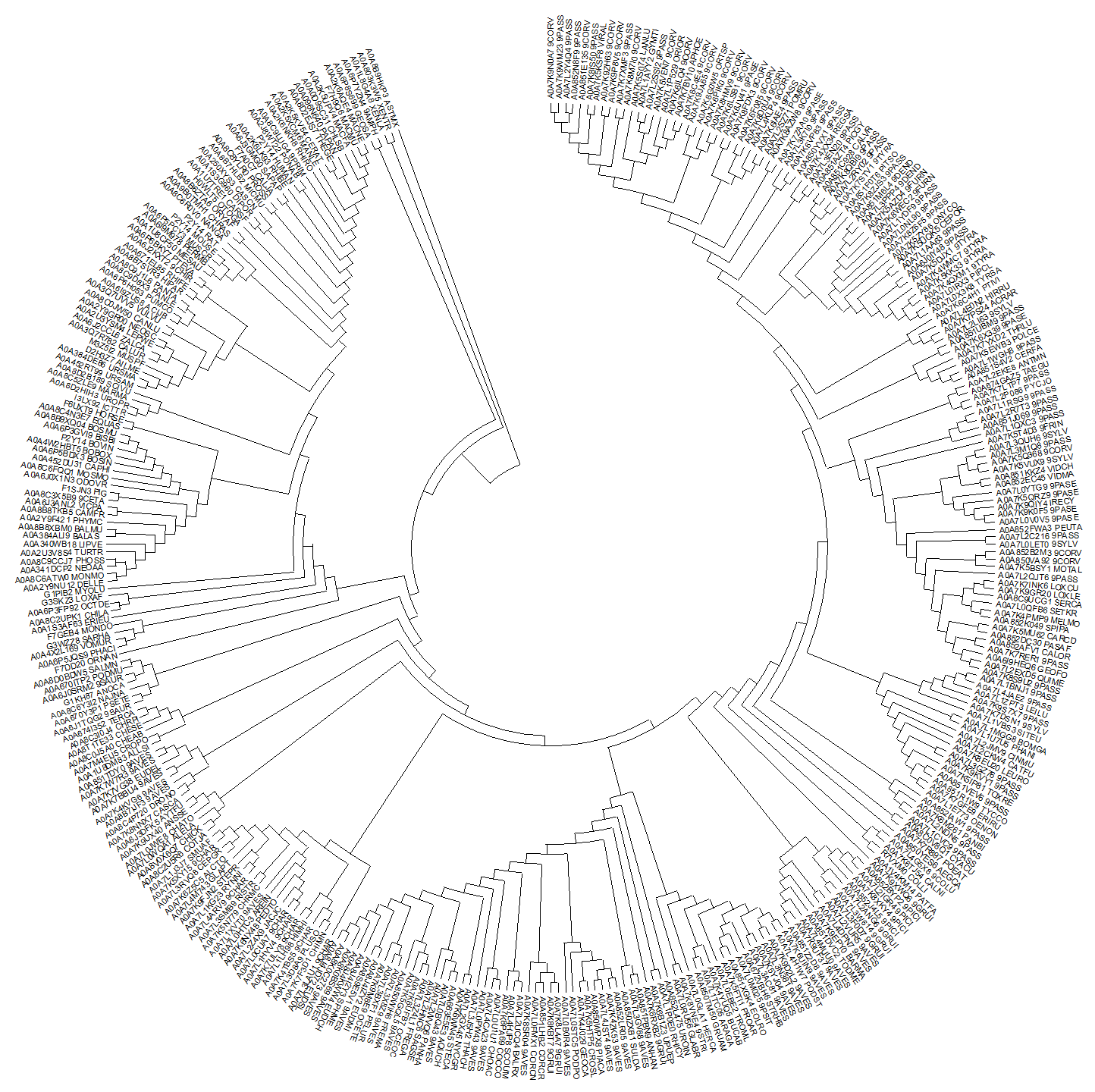


**Figure 4*—*source data 4.** The neighbor joining tree of P2Y14 showing evolutionary range of species.

Supplement: Figure 4—source data 4. [file elife-85449-fig4-data4.docx]

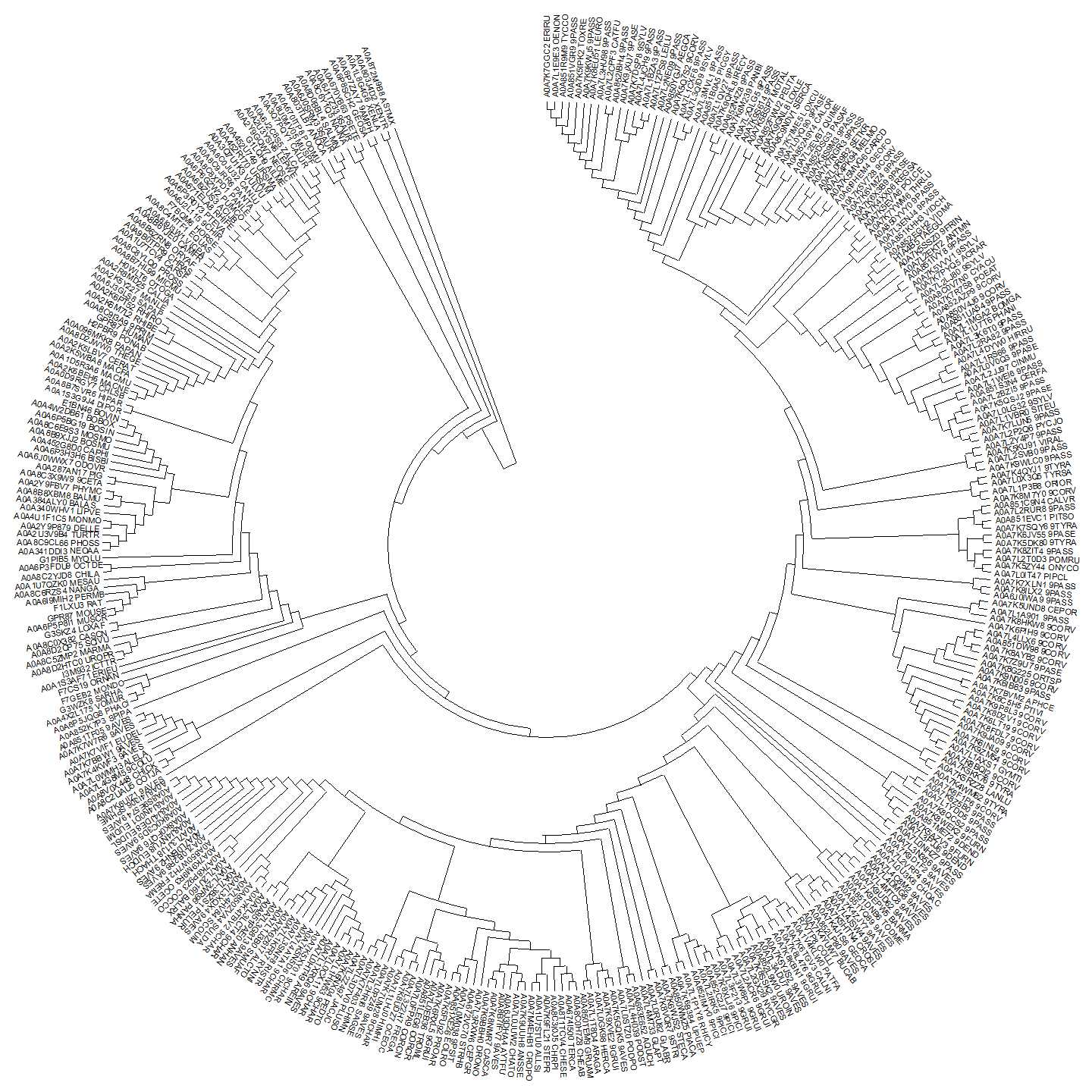


**Figure 4*—*source data 5.** The neighbor joining tree of GPR87 showing evolutionary range of species.

Supplement: Figure 4—source data 5. [file elife-85449-fig4-data5.docx]
