# Supplementary material for: Identification of a carbohydrate recognition motif of purinergic receptors
Source: eLife. 2023 Nov 13;12:e85449. doi: 10.7554/eLife.85449 (PMC10642967; doi:10.7554/eLife.85449)
Supplement: Supplementary file 1. [file elife-85449-supp1.docx]

**Supplementary file 1a.** Expression of mutants compared with WT P2Y14 in HEK293.

| **Construct** | **Expression (%WT)** | **Comment** |
| --- | --- | --- |
| WT | 100 |  |
| K77A | 36.3 ± 2.5 **** | < 40.0 |
| D81A | 57.0 ± 2.7 **** |  |
| N90A | 9.9 ± 1.0 **** | < 40.0 |
| I170A | 66.5 ± 6.4 ** |  |
| R253A | 84.1 ± 4.8* |  |
| T257A | 85.1 ± 7.2 |  |
| R274A | 95.3 ± 4.5 |  |
| R274F | 97.4 ± 0.4 |  |
| K277A | 33.5 ± 2.8**** | < 40.0 |
| E278A | 51.8 ± 3.2 **** |  |

Data are shown as means ± SEM of at least three independent experiments performed duplicate. Unpaired t test was used to determine statistical difference; **P* < 0.05, ***P* < 0.01, *****P* < 0.0001.

**Supplementary file 1b.** Expression of mutants compared with WT P2Y12 in HEK293.

| **Construct** | **Expression (%WT)** | **Comment** |
| --- | --- | --- |
| WT | 100 |  |
| K80A | 65.4 ± 3.0 **** |  |
| D84A | 72.2 ± 2.0 **** |  |
| F277A | 94.4 ± 3.8 |  |
| K280A | 69.6 ± 2.4 **** |  |
| E281A | 76.5 ± 2.3 **** |  |

Data are shown as means ± SEM of at least three independent experiments performed duplicate. Unpaired t test was used to determine statistical difference; *****P* < 0.0001.
