# Supplementary material for: Identification of a carbohydrate recognition motif of purinergic receptors
Source: eLife. 2023 Nov 13;12:e85449. doi: 10.7554/eLife.85449 (PMC10642967; doi:10.7554/eLife.85449)
Supplement: Supplementary file 2. [file elife-85449-supp2.docx]

**Supplementary file 2a.** Average distance between atom pairs in apo and UDP-Glc-bound P2Y14 simulations.

| Atom pairs | Distance  in apo P2Y14  *d_apo_* (Å) | Distance in  UDP-Glc-bound P2Y14  *d_liganded_* (Å) | *d_apo_* – *d_liganded_* (Å) | Comment |
| --- | --- | --- | --- | --- |
| Q88^3.19^ Cα and S259^6.61^ Cα | 29.2 ± 1.3 | 25.8 ± 0.6 | 3.4 | Helical distance at extracellular side of TM3 and TM6 |
| Q88^3.19^ Cα and C267^7.25^ Cα | 29.1 ± 1.4 | 25.0 ± 0.9 | 4.1 | Helical distance at extracellular side of TM3 and TM7 |
| W183^5.35^ Cα and S259^6.61^ Cα | 21.1 ± 1.2 | 15.2 ± 0.4 | 5.9 | Helical distance at Extracellular side of TM5 and TM6 |
| Y189^5.41^ sidechain carbon and T257^6.59^ sidechain carbon | 8.5 ± 1.0 | 3.8 ± 0.5 | 4.7 | Minimal distance between residues of TM5 and TM6 |
| K277^7.35^ sidechain nitrogen and UDP-Glc glucose 6’ hydroxyl group | Not applicable | 2.6 ± 0.1 | Not applicable | Hydrogen bonding between K277 and UDP-Glc glucose 6’ hydroxyl group |
| E278^7.36^ sidechain oxygen and UDP-Glc glucose 6’ hydroxyl group | Not applicable | 2.8 ± 0.4 | Not applicable | Hydrogen bonding between E278^7.36^ sidechain oxygen and UDP-Glc glucose 6’ hydroxyl group |
| R253^6.55^ sidechain nitrogen and UDP-Glc phosphate group | Not applicable | 2.7 ± 0.1 | Not applicable | Salt bridging between R253^6.55^ sidechain nitrogen and UDP-Glc phosphate group |

**Supplementary file 2b.** Average distance between atom pairs in UDP-Glc-bound, UDP-GlcA-bound, UDP-GlcNAc-bound and UDP-Gal-bound P2Y14 simulations.

| Atom pairs | Distance in  UDP-Glc-  bound  P2Y14 (Å) | Distance in  UDP-GlcA-bound  P2Y14 (Å) | Distance in  UDP-GlcNAc-bound  P2Y14 (Å) | Distance in  UDP-Gal-  bound  P2Y14 (Å) | Comment |
| --- | --- | --- | --- | --- | --- |
| K77^2.60^ sidechain nitrogen  and D81^2.64^ sidechain oxygen | 2.8 ± 0.3 | 2.8 ± 0.1 | 2.7 ± 0.1 | 2.7 ± 0.1 | Salt bridge |
| K77^2.60^ sidechain nitrogen  and E278^7.36^ sidechain oxygen | 2.7 ± 0.1 | 2.8 ± 0.1 | 2.7 ± 0.1 | 2.7 ± 0.2 | Salt bridge |
| K277^7.35^ sidechain nitrogen  and E278^7.36^ sidechain oxygen | 2.7 ± 0.2 | 2.7 ± 0.1 | 2.7 ± 0.1 | 2.7 ± 0.1 | Salt bridge |

**Supplementary file 2c.** The percentage of hydrogen bonding occurrence between a residue of P2Y14 and a ligand in MD simulations.

| **Ligand** | **Residue** | **Percentage of occurrence of hydrogen bonding** |
| --- | --- | --- |
| UDP-Glc | K277 | 99.8% |
| UDP-GlcA | K277 | 100.0% |
| UDP-GlcNAc | K277 | 81.0% |
| UDP-Gal | K277 | 100.0% |
| UDP-Glc | E278 | 83.4% |
| UDP-GlcA | E278 | 0.0% |
| UDP-GlcNAc | E278 | 1.0% |
| UDP-Gal | E278 | 11.6% |
| UDP-Glc | R253 | 100% |
| UDP-GlcA | R253 | 83.4% |
| UDP-GlcNAc | R253 | 99.1% |
| UDP-Gal | R253 | 99.9% |
| UDP-Glc | K77 | 72.8% |
| UDP-GlcA | K77 | 92.2% |
| UDP-GlcNAc | K77 | 47.9% |
| UDP-Gal | K77 | 80.6% |
