# Supplementary material for: Identification of a carbohydrate recognition motif of purinergic receptors
Source: eLife. 2023 Nov 13;12:e85449. doi: 10.7554/eLife.85449 (PMC10642967; doi:10.7554/eLife.85449)
Supplement: Supplementary file 3. [file elife-85449-supp3.docx]

**Supplementary file 3a.** Species repertoire information for P2Y12, P2Y13, P2Y14 and GPR87.

| **Number** | **Species** | **UniProt ID** | | | |
| --- | --- | --- | --- | --- | --- |
|  |  | **P2Y12** | **P2Y13** | **P2Y14** | **GPR87** |
| 1 | Acinonyx jubatus | A0A6I9ZSX5 | A0A6I9ZU69 | A0A6I9ZUS8 | A0A6I9ZUS3 |
| 2 | Acrocephalus arundinaceus | A0A7K7Q3K4 | A0A7K7PY85 | A0A7K7PS24 | A0A7K7PYQ5 |
| 3 | Aegithalos caudatus | A0A850YHP8 | A0A850YI23 | A0A850YES6 | A0A850YGJ7 |
| 4 | Aegotheles bennettii | A0A7K6UHF3 | A0A7K6UIP6 | A0A7K6UIN9 | A0A7K6UIZ1 |
| 5 | Ailuropoda melanoleuca | G1MLB2 | G1ML91 | D2H3Z7 | G1LQH9 |
| 6 | Alaudala cheleensis | A0A7L2BXD8 | A0A7L2C0U9 | A0A7L2C216 | A0A7L2BZI5 |
| 7 | Alca torda | A0A7K6Z5D4 | A0A7K6Z4L5 | A0A7K6Z5C5 | A0A7K6Z617 |
| 8 | Aleadryas rufinucha | A0A7K8HK57 | A0A7K8HK25 | A0A7K8HMV9 | A0A7K8HKW8 |
| 9 | Alectura lathami | A0A7L0WL40 | A0A7L0WMP9 | A0A7L0WQA1 | A0A7L0WMH3 |
| 10 | Alligator sinensis | A0A1U7SLD4 | A0A1U7SWD7 | A0A1U8DM83 | A0A1U7STU0 |
| 11 | Alopecoenas beccarii | A0A7L4G636 | A0A7L4G6A8 | A0A7L4G5X8 | A0A7L4G8M5 |
| 12 | Amazona guildingii | A0A7L0LZY3 | A0A7L0LZS5 | A0A7L0M025 | A0A7L0M038 |
| 13 | Anhinga anhinga | A0A851PKD2 | A0A851PJ02 | A0A851PBN9 | A0A851PAE0 |
| 14 | Anhinga rufa | A0A7L3GNP1 | A0A7L3GND5 | A0A7L3GV68 | A0A7L3G813 |
| 15 | Anolis carolinensis | G1KWB7 | G1KUN3 | G1KH87 | A0A803TLB7 |
| 16 | Anseranas semipalmata | A0A7K9UYI9 | A0A7K9UWN5 | A0A7K9UX40 | A0A7K9UUH8 |
| 17 | Anthoscopus minutus | A0A7L2EKD3 | A0A7L2EL18 | A0A7L2EKE8 | A0A7L2EKT2 |
| 18 | Aphelocoma coerulescens | A0A7K7C5R3 | A0A7K7BW49 | A0A7K7BV10 | A0A7K7BVM2 |
| 19 | Apteryx mantelli mantelli | A0A8B7IFY3 | A0A8B7IJF8 | A0A8B7IJF3 | A0A8B7IFV7 |
| 20 | Aquila chrysaetos chrysaetos | A0A663E5K8 | A0A663E610 | A0A663E5E3 | A0A663E652 |
| 21 | Aramus guarauna | A0A7L1T2Y9 | A0A7L1T2Y6 | A0A7L1TC05 | A0A7L1T8D4 |
| 22 | Ardeotis kori | A0A7K8L461 | A0A7K8L3V1 | A0A7K8L4A7 | A0A7K8L476 |
| 23 | Arenaria interpres | A0A7L0HSB4 | A0A7L0HSC3 | A0A7L0HTC3 | A0A7L0HT49 |
| 24 | Astyanax mexicanus | A0A8T2M392 | A0A8B9L4C8 | A0A8B9HKP3 | A0A8T2M9B8 |
| 25 | Atlantisia rogersi | A0A7L3W4R4 | A0A7L3W7K2 | A0A7L3W614 | A0A7L3W8P1 |
| 26 | Aythya fuligula | A0A6J3DBS1 | A0A6J3DHU6 | A0A6J3DFK5 | A0A6J3DHA4 |
| 27 | Balaeniceps rex | A0A7L2U0Z9 | A0A7L2U0P4 | A0A7L2UCQ4 | A0A7L2U160 |
| 28 | Balaenoptera acutorostrata scammoni | A0A384BH24 | A0A384ALI6 | A0A384ALI9 | A0A384ALY0 |
| 29 | Balaenoptera musculus | A0A8B8XG87 | A0A8B8XB87 | A0A8B8XBM0 | A0A8B8XBM8 |
| 30 | Baryphthengus martii | A0A7K9EPZ1 | A0A7K9EQN3 | A0A7K9EPY0 | A0A7K9EPW5 |
| 31 | Bison bison bison | A0A6P3H3R4 | A0A6P3GQ99 | A0A6P3GVI9 | A0A6P3H3H6 |
| 32 | Bombycilla garrulus | A0A7L1MFW0 | A0A7L1MI21 | A0A7L1MGG8 | A0A7L1MGA2 |
| 33 | Bos indicus | A0A6P5BT09 | A0A6P5BT20 | A0A6P5BDX3 | A0A6P5BG19 |
| 34 | Bos indicus x Bos taurus | A0A4W2DVS5 | A0A4W2FFQ5 | A0A4W2HBT5 | A0A4W2DB61 |
| 35 | Bos mutus grunniens | A0A8B9XJ76 | A0A8B9XHU5 | A0A8B9XQ04 | A0A8B9XJJ2 |
| 36 | Bos taurus | Q0IIL0 | A5D7I6 | Q3SX17 | E1BN46 |
| 37 | Brachypodius atriceps | A0A7K7LUE3 | A0A7K7LTA7 | A0A7K7LTP7 | A0A7K7LUN5 |
| 38 | Brachypteracias leptosomus | A0A7L2VUL1 | A0A7L2VWK8 | A0A7L2VUR5 | A0A7L2VRP4 |
| 39 | Bucorvus abyssinicus | A0A7K4YVC4 | A0A7K4YUY8 | A0A7K4YUQ5 | A0A7K4YUW7 |
| 40 | Buphagus erythrorhynchus | A0A7L3HP95 | A0A7L3HQI5 | A0A7L3GZ76 | A0A7L3HU98 |
| 41 | Burhinus bistriatus | A0A7K4T999 | A0A7K4T9B6 | A0A7K4TBS5 | A0A7K4TBV2 |
| 42 | Calcarius ornatus | A0A852AGA9 | A0A852AA49 | A0A852AFV1 | A0A852A9Y4 |
| 43 | Callaeas wilsoni | A0A7L4KVU8 | A0A7L4LLX1 | A0A7L4KLP4 | A0A7L4LLX6 |
| 44 | Callithrix jacchus | U3DDL8 | F6XPZ9 | F7A0C7 | A0A2R8MD25 |
| 45 | Callorhinus ursinus | A0A3Q7Q1W1 | A0A3Q7Q1S4 | A0A3Q7R782 | A0A3Q7PQY1 |
| 46 | Caloenas nicobarica | A0A7K6TH83 | A0A7K6TJC8 | A0A7K6TJ54 | A0A7K6TGV3 |
| 47 | Calonectris borealis | A0A7L3XRP1 | A0A7L3XQA0 | A0A7L3X8Z9 | A0A7L3XQX4 |
| 48 | Calyptomena viridis | A0A851C7L4 | A0A851CA86 | A0A851C928 | A0A851C9N4 |
| 49 | Camelus ferus | A0A8B7KCT8 | A0A8B6YJB3 | A0A8B8TKB5 | A0A8B6YJE0 |
| 50 | Campylorhamphus procurvoides | A0A851MFH1 | A0A851M6Q9 | A0A851M6L4 | A0A851ME72 |
| 51 | Canis lupus dingo | A0A8C0JT64 | A0A8C0JWW5 | A0A8C0JW50 | A0A8C0JU32 |
| 52 | Capra hircus | A0A452FKL2 | A0A452DVY1 | A0A452DU31 | A0A452G8D0 |
| 53 | Cardinalis cardinalis | A0A7K5MU73 | A0A7K5MUZ9 | A0A7K5MU62 | A0A7K5MVG6 |
| 54 | Carlito syrichta | A0A1U7TRG7 | A0A1U7T6I4 | A0A1U7TRE1 | A0A1U7TCV4 |
| 55 | Castor canadensis | A0A250YG01 | A0A250YFM7 | A0A250XYS3 | A0A8C0X382 |
| 56 | Casuarius casuarius | A0A7K8NN30 | A0A7K8NMT0 | A0A7K8NNX7 | A0A7K8NMR7 |
| 57 | Catagonus wagneri | A0A8C3X182 | A0A8C3X9Z2 | A0A8C3X5B9 | A0A8C3X9W9 |
| 58 | Catharus fuscescens | A0A7L2CPF8 | A0A7L2CPF9 | A0A7L2CKW4 | A0A7L2CPF3 |
| 59 | Centropus bengalensis | A0A852LU92 | A0A852M015 | A0A852LR05 | A0A852LP80 |
| 60 | Centropus unirufus | A0A7K4ZJS5 | A0A7K4ZJS2 | A0A7K4ZK53 | A0A7K4ZJY7 |
| 61 | Cephalopterus ornatus | A0A7K5URY5 | A0A7K5UN51 | A0A7K5UQK5 | A0A7K5UND8 |
| 62 | Cepphus grylle | A0A7L3RVG2 | A0A7L3RYK2 | A0A7L3RVQ8 | A0A7L3RXW6 |
| 63 | Cercocebus atys | A0A2K5L384 | A0A2K5MTQ6 | A0A2K5KV54 | A0A2K5LBV7 |
| 64 | Certhia brachydactyla | A0A7L1WEJ6 | A0A7L1WEI9 | A0A7L1WGH8 | A0A7L1WEI6 |
| 65 | Certhia familiaris | A0A851SCS8 | A0A851SC25 | A0A851S4V2 | A0A851S3N4 |
| 66 | Cettia cetti | A0A7L3PZH2 | A0A7L3Q144 | A0A7L3QUH6 | A0A7L3QID9 |
| 67 | Ceuthmochares aereus | A0A7L4JSR0 | A0A7L4JSS5 | A0A7L4JST4 | A0A7L4JSW4 |
| 68 | Ceyx cyanopectus | A0A7L4NQE5 | A0A7L4NQW9 | A0A7L4MUU0 | A0A7L4MTC8 |
| 69 | Chaetops frenatus | A0A7L3EL82 | A0A7L3EQS9 | A0A7L3EN20 | A0A7L3ENJ4 |
| 70 | Chaetorhynchus papuensis | A0A7K8IP00 | A0A7K8IMR0 | A0A7K8IS50 | A0A7K8ILX2 |
| 71 | Chauna torquata | A0A7L0JVP4 | A0A7L0JUW0 | A0A7L0JWE8 | A0A7L0JUW2 |
| 72 | Chelonoidis abingdonii | A0A8C0IRB8 | A0A8C0GW15 | A0A8C0J5A0 | A0A8C0H7Z8 |
| 73 | Chelydra serpentina | A0A8C3SYL7 | A0A8T1TEQ0 | A0A8T1TE33 | A0A8T1TCV4 |
| 74 | Chinchilla lanigera | A0A8C2URI8 | A0A8C2UQI2 | A0A8C2UPK1 | A0A8C2YJD8 |
| 75 | Chionis minor | A0A7K7F2X0 | A0A7K7F380 | A0A7K7F3D1 | A0A7K7F403 |
| 76 | Chlorocebus sabaeus | A0A0D9SC29 | A0A0D9RGZ4 | A0A0D9SC31 | A0A0D9RGY7 |
| 77 | Chloroceryle aenea | A0A7K9U4V4 | A0A7K9UD76 | A0A7K9UF37 | A0A7K9UD62 |
| 78 | Chloropsis cyanopogon | A0A852AF78 | A0A852AM51 | A0A852B2M3 | A0A852AZP9 |
| 79 | Chloropsis hardwickii | A0A850V4T7 | A0A850V2S8 | A0A850VA92 | A0A850V4J6 |
| 80 | Chordeiles acutipennis | A0A7L0U4P5 | A0A7L0U1Q5 | A0A7L0U1U1 | A0A7L0U3K6 |
| 81 | Chroicocephalus maculipennis | A0A7K5N4W2 | A0A7K5N599 | A0A7K5N779 | A0A7K5N5D6 |
| 82 | Chrysemys picta bellii | A0A8C3P9T2 | A0A8C3I0D9 | A0A8C3I0J4 | A0A8C3I0J5 |
| 83 | Chrysochloris asiatica | A0A9B0TUP7 | A0A9B0TNJ4 | A0A9B0TMH1 | A0A9B0TPR9 |
| 84 | Chunga burmeisteri | A0A7K5GQJ8 | A0A7K5GQQ4 | A0A7K5GQL5 | A0A7K5GQK5 |
| 85 | Ciccaba nigrolineata | A0A7K8VND9 | A0A7K8VR04 | A0A7K8VNE4 | A0A7K8VQR7 |
| 86 | Ciconia maguari | A0A7L0B534 | A0A7L0B0Q6 | A0A7L0B0R4 | A0A7L0B2R6 |
| 87 | Cinclus mexicanus | A0A7L2JUK4 | A0A7L2JJ78 | A0A7L2JMV9 | A0A7L2JJ97 |
| 88 | Cisticola juncidis | A0A7L1QXA3 | A0A7L1QXA6 | A0A7L1QXC3 | A0A7L1QV27 |
| 89 | Cnemophilus loriae | A0A7K8AY71 | A0A7K8AY68 | A0A7K8AZW8 | A0A7K8AYB2 |
| 90 | Cochlearius cochlearius | A0A7K8P9X8 | A0A7K8PA45 | A0A7K8PA69 | A0A7K8P9Z3 |
| 91 | Columba livia | A0A2I0M1M5 | A0A2I0M1J5 | R7VXM0 | R7VTP8 |
| 92 | Copsychus sechellarum | A0A851VGS4 | A0A851VER7 | A0A851VEV6 | A0A851VGR9 |
| 93 | Corythaeola cristata | A0A851L8Q1 | A0A851L8V4 | A0A851LHB2 | A0A851LEG6 |
| 94 | Corythaixoides concolor | A0A7L0FMC0 | A0A7L0FMZ0 | A0A7L0FMX1 | A0A7L0FPA9 |
| 95 | Coturnix japonica | A0A8C2YFY9 | A0A8C2U7H6 | A0A8C2U5R6 | A0A8C2UAU5 |
| 96 | Crocodylus porosus | A0A7M4EHB3 | A0A7M4EH64 | A0A7M4EIJ5 | A0A7M4EHB1 |
| 97 | Crotophaga sulcirostris | A0A7K5HT90 | A0A7K5HTM0 | A0A7K5HTP5 | A0A7K5HTN4 |
| 98 | Crypturellus soui | A0A7K4KWC6 | A0A7K4KWD4 | A0A7K4KVG6 | A0A7K4KWF3 |
| 99 | Cyanistes caeruleus | A0A8C0V6V0 | A0A8C0V203 | A0A8C0V8Q1 | A0A8C0V7N0 |
| 100 | Daphoenositta chrysoptera | A0A7K6F180 | A0A7K6F2U4 | A0A7K6F085 | A0A7K6F1P6 |
| 101 | Dasyornis broadbenti | A0A7K6ICF6 | A0A7K6IBU5 | A0A7K6IAE4 | A0A7K6IB63 |
| 102 | Delphinapterus leucas | A0A2Y9NZS7 | A0A2Y9NU17 | A0A2Y9NU12 | A0A2Y9P879 |
| 103 | Dicaeum eximium | A0A7K9K0J4 | A0A7K9K0K4 | A0A7K9K0F5 | A0A7K9JXU7 |
| 104 | Dicrurus megarhynchus | A0A7K9ZK87 | A0A7K9ZH82 | A0A7K9ZH63 | A0A7K9ZM54 |
| 105 | Dipodomys ordii | A0A1S3G972 | A0A1S3GAU2 | A0A1S3G960 | A0A1S3G9J4 |
| 106 | Donacobius atricapilla | A0A851J082 | A0A851J1W0 | A0A851J069 | A0A851IVY8 |
| 107 | Dromaius novaehollandiae | A0A7K9BA73 | A0A8C4JD44 | A0A8C4P720 | A0A7K9BBH0 |
| 108 | Dromas ardeola | A0A7K5XY28 | A0A7K5XY68 | A0A7K5XY15 | A0A7K5XY01 |
| 109 | Drymodes brunneopygia | A0A7L3K8D1 | A0A7L3K878 | A0A7L3K7I0 | A0A7L3K6T0 |
| 110 | Dryoscopus gambensis | A0A851E2C8 | A0A851E130 | A0A851E135 | A0A851DW98 |
| 111 | Edolisoma coerulescens | A0A7K9N0E0 | A0A7K9N009 | A0A7K9N0A7 | A0A7K9N005 |
| 112 | Elachura formosa | A0A851UB37 | A0A851UB55 | A0A851UBM9 | A0A851UA54 |
| 113 | Eolophus roseicapilla | A0A851XDG9 | A0A851XGM6 | A0A851XGK4 | A0A851XE26 |
| 114 | Equus asinus asinus | A0A8C4MVH7 | A0A8C4MUU3 | A0A8C4N3E7 | A0A8C4MTF1 |
| 115 | Equus caballus | F6T4Y5 | F6TM75 | F6UXT9 | F7BQM6 |
| 116 | Erinaceus europaeus | A0A1S3AF23 | A0A1S3AEW0 | A0A1S3AF63 | A0A1S3AF71 |
| 117 | Erithacus rubecula | A0A7K7GG63 | A0A7K7GFH6 | A0A7K7GFE9 | A0A7K7GGC2 |
| 118 | Erpornis zantholeuca | A0A7L2Y2R7 | A0A7L2Y806 | A0A7L2Y4Q4 | A0A7L2Y4P7 |
| 119 | Erythrocercus mccallii | A0A7K5Q7Q8 | A0A7K5Q6Z7 | A0A7K5Q368 | A0A7K5Q7S2 |
| 120 | Eubucco bourcierii | A0A7K8XMQ4 | A0A7K8XLK2 | A0A7K8XKY4 | A0A7K8XLJ6 |
| 121 | Eudromia elegans | A0A7K7VG14 | A0A7K7VG37 | A0A7K7VG38 | A0A7K7VIF1 |
| 122 | Eudyptes chrysocome | A0A8J4KWT6 | A0A8J4KY50 | A0A8J4KWW4 | A0A8J4NYU8 |
| 123 | Eudyptes filholi | A0A8K0BSE1 | A0A8K0FQV9 | A0A8K0BS69 | A0A8K0FQT6 |
| 124 | Eudyptes robustus | A0A8K0FWI9 | A0A8K0C2X8 | A0A8K0C273 | A0A8K0C3D5 |
| 125 | Eudyptes sclateri | A0A8J4JEJ5 | A0A8J4JD57 | A0A8J4JD72 | A0A8J4JDE8 |
| 126 | Eudyptula minor | A0A8J4IZT2 | A0A8J4N0G8 | A0A8J4IZY2 | A0A8J4N0D1 |
| 127 | Eudyptula novaehollandiae | A0A8S9E5M6 | A0A8S9E5S3 | A0A8S9E5V1 | A0A8S9E574 |
| 128 | Eulacestoma nigropectus | A0A7K8D0W2 | A0A7K8D4K1 | A0A7K8D0U4 | A0A7K8D2V1 |
| 129 | Eurystomus gularis | A0A7L4DLQ6 | A0A7L4DN85 | A0A7L4DPN7 | A0A7L4DMG8 |
| 130 | Falcunculus frontatus | A0A7K6LT45 | A0A7K6LR88 | A0A7K6LSB1 | A0A7K6LT19 |
| 131 | Formicarius rufipectus | A0A7L0NJB8 | A0A7L0NI08 | A0A7L0NL90 | A0A7L0NHZ7 |
| 132 | Fregata magnificens | A0A850WCE9 | A0A850W6X4 | A0A850WIH6 | A0A850W7H2 |
| 133 | Fregetta grallaria | A0A7L3Z2T3 | A0A7L3Z1S7 | A0A7L3Z4L1 | A0A7L3Z2H7 |
| 134 | Furnarius figulus | A0A7K5B0U3 | A0A7K5AZB5 | A0A7K5AZD4 | A0A7K5AZV3 |
| 135 | Gallus gallus | A0A8V0X9C4 | A0A8V0X443 | A0A8V0X6Q7 | A0A8V0X448 |
| 136 | Geococcyx californianus | A0A7K4J058 | A0A7K4IZW4 | A0A7K4J029 | A0A7K4J1S6 |
| 137 | Geospiza fortis | A0A6I9HEQ0 | A0A6I9HES8 | A0A6I9HEQ6 | A0A6I9HEM4 |
| 138 | Geotrypetes seraphini | A0A6P8SAL7 | A0A6P8S6X0 | A0A6P8S699 | A0A6P8SCH2 |
| 139 | Glareola pratincola | A0A7L4M770 | A0A7L4MA48 | A0A7L4M743 | A0A7L4M733 |
| 140 | Glaucidium brasilianum | A0A7L0RTZ7 | A0A7L0RWN7 | A0A7L0RU56 | A0A7L0RU82 |
| 141 | Grallaria varia | A0A7K8ZLH9 | A0A7K8ZIP3 | A0A7K8ZJS3 | A0A7K8ZIT4 |
| 142 | Grus americana | A0A850TIN0 | A0A850TAL3 | A0A850TM50 | A0A850TEM9 |
| 143 | Gymnorhina tibicen | A0A7L1B8F7 | A0A7L1AY11 | A0A7L1AYY2 | A0A7L1AXS1 |
| 144 | Halcyon senegalensis | A0A851YX32 | A0A851Z2C8 | A0A851Z2D8 | A0A851YQ89 |
| 145 | Heliornis fulica | A0A7L2AI10 | A0A7L2ACQ6 | A0A7L2ANG6 | A0A7L2ACR6 |
| 146 | Hemiprocne comata | A0A7K9D1F1 | A0A7K9D2Y2 | A0A7K9D2L2 | A0A7K9D1F2 |
| 147 | Herpetotheres cachinnans | A0A7L0GKG4 | A0A7L0GKH6 | A0A7L0GLA1 | A0A7L0GK98 |
| 148 | Himantopus himantopus | A0A7L1LH71 | A0A7L1LHT1 | A0A7L1LH98 | A0A7L1LHJ0 |
| 149 | Hippolais icterina | A0A7L2LJF2 | A0A7L2LIN9 | A0A7L2LI63 | A0A7L2LJ80 |
| 150 | Hipposideros armiger | A0A8B7STZ7 | A0A8B7STZ6 | A0A8B7SVR3 | A0A8B7SVR6 |
| 151 | Hirundo rustica | A0A7L4DYE4 | A0A7L4DYQ2 | A0A7L4E0N2 | A0A7L4DYW0 |
| 152 | Homo sapiens | Q9H244 | Q9BPV8 | Q15391 | Q9BY21 |
| 153 | Horornis vulcanius | A0A7L3MPK3 | A0A7L3MQI5 | A0A7L3M1Q8 | A0A7L3MVL1 |
| 154 | Hylia prasina | A0A7K5W421 | A0A7K5W2Y3 | A0A7K5VUX9 | A0A7K5VVV1 |
| 155 | Hypocryptadius cinnamomeus | A0A7L2QCH0 | A0A7L2QLY6 | A0A7L2QJT6 | A0A7L2QLG5 |
| 156 | Ibidorhyncha struthersii | A0A7K7UNV9 | A0A7K7ULE6 | A0A7K7ULY8 | A0A7K7UM28 |
| 157 | Ictidomys tridecemlineatus | I3MFN5 | I3NDA8 | I3LX92 | I3M932 |
| 158 | Ifrita kowaldi | A0A7K6PCM5 | A0A7K6PCM8 | A0A7K6PIK0 | A0A7K6PIH9 |
| 159 | Illadopsis cleaveri | A0A7L1BMC3 | A0A7L1BM40 | A0A7L1BNJ1 | A0A7L1BZA3 |
| 160 | Irena cyanogastra | A0A7K9QGZ9 | A0A7K9QH46 | A0A7K9QIY4 | A0A7K9QHL8 |
| 161 | Jacana jacana | A0A7L2Z487 | A0A7L2Z2S8 | A0A7L2Z4X9 | A0A7L2Z497 |
| 162 | Lanius ludovicianus | A0A7K5RXI4 | A0A7K5RXW2 | A0A7K5S074 | A0A7K5RZZ8 |
| 163 | Leiothrix lutea | A0A7L1ZVD3 | A0A7L1ZPT7 | A0A7L1ZPT3 | A0A7L1ZPS8 |
| 164 | Lepidothrix coronata | A0A6J0IWD2 | A0A6J0IY96 | A0A6J0IY48 | A0A6J0IWA9 |
| 165 | Leptocoma aspasia | A0A7L0V0S4 | A0A7L0V0Q7 | A0A7L0V0V5 | A0A7L0V0Q3 |
| 166 | Leptonychotes weddellii | A0A2U3Z4D6 | A0A7F8Q9N8 | A0A2U3YSM4 | A0A2U3YSN6 |
| 167 | Leucopsar rothschildi | A0A7K8EU19 | A0A7K8EU57 | A0A7K8EU20 | A0A7K8EU51 |
| 168 | Lipotes vexillifer | A0A340WDN6 | A0A340WI50 | A0A340WB18 | A0A340WHV1 |
| 169 | Locustella ochotensis | A0A7L1RS35 | A0A7L1RZ61 | A0A7L1RSG9 | A0A7L1RS66 |
| 170 | Lophotis ruficrista | A0A7K8KBM9 | A0A7K8KCC9 | A0A7K8KBT7 | A0A7K8KBN1 |
| 171 | Loxia curvirostra | A0A7K7IMG2 | A0A7K7INB7 | A0A7K7INK6 | A0A7K7IME5 |
| 172 | Loxia leucoptera | A0A7K9GNS6 | A0A7K9GNK7 | A0A7K9GR20 | A0A7K9GNL8 |
| 173 | Loxodonta africana | G3TP25 | G3TQA0 | G3SKZ3 | G3SKZ4 |
| 174 | Macaca fascicularis | Q95KC3 | Q9BE53 | A0A2K5UPV4 | A0A2K5WBA8 |
| 175 | Macaca mulatta | F6PW20 | A0A1D5Q1M0 | F7H9Q6 | A0A1D5R3A6 |
| 176 | Macaca nemestrina | A0A2K6ANJ8 | A0A2K6ATF5 | A0A2K6AQE4 | A0A2K6BEH6 |
| 177 | Machaerirhynchus nigripectus | A0A7K6ILQ5 | A0A7K6IMS2 | A0A7K6ILQ4 | A0A7K6INL9 |
| 178 | Mandrillus leucophaeus | A0A2K5XP54 | A0A2K5ZJZ5 | A0A2K5XCM6 | A0A2K5Y227 |
| 179 | Marmota marmota marmota | A0A8C6EV09 | A0A8C5ZM32 | A0A8C5ZLE9 | A0A8C5ZMP2 |
| 180 | Melanocharis versteri | A0A7K7Z9J4 | A0A7K7ZC97 | A0A7K7ZAA0 | A0A7K7Z9U7 |
| 181 | Melospiza melodia | A0A7K4PK74 | A0A7K4PK50 | A0A7K4PMP9 | A0A7K4PK94 |
| 182 | Mesembrinibis cayennensis | A0A7L0PY65 | A0A7L0PX75 | A0A7L0PW43 | A0A7L0PZ49 |
| 183 | Mesocricetus auratus | A0A1U7QFK2 | A0A1U7QP40 | A0A1U8CF50 | A0A1U7QZK0 |
| 184 | Microcaecilia unicolor | A0A6P7ZA63 | A0A6P7Z578 | A0A6P7YZN4 | A0A6P7ZAY7 |
| 185 | Microcebus murinus | A0A8B7X0W2 | A0A8B7HLA3 | A0A8B7HLB2 | A0A8B7HL99 |
| 186 | Mionectes macconnelli | A0A7K5KKW7 | A0A7K5KK31 | A0A7K5KK33 | A0A7K5KK76 |
| 187 | Mohoua ochrocephala | A0A7K7XLF7 | A0A7K7XMR1 | A0A7K7XMF3 | A0A7K7XLN1 |
| 188 | Monodelphis domestica | F7DAY4 | A0A5F8GXU1 | F7GEB4 | F7GEB2 |
| 189 | Monodon monoceros | A0A8C6ARL6 | A0A8C6F2M0 | A0A8C6ATW0 | A0A4U1F1C5 |
| 190 | Moschus moschiferus | A0A8C6E1L3 | A0A8C6E0U3 | A0A8C6FQQ1 | A0A8C6E9S3 |
| 191 | Motacilla alba | A0A7K5BUC7 | A0A7K5BSQ9 | A0A7K5BSY1 | A0A7K5BSP7 |
| 192 | Mus caroli | A0A6P7QRC9 | A0A6P5PEM6 | A0A6P5PCY3 | A0A6P5P8I1 |
| 193 | Mus musculus | Q9CPV9 | Q9D8I2 | Q9ESG6 | Q99MT7 |
| 194 | Mustela putorius furo | M3Z5J3 | M3Z5I8 | M3Z5I2 | A0A8U0MV05 |
| 195 | Myiagra hebetior | A0A7K9JBY0 | A0A7K9JBA5 | A0A7K9JA65 | A0A7K9JA09 |
| 196 | Myotis lucifugus | G1PID3 | G1PIC3 | G1PIB2 | G1PIB5 |
| 197 | Mystacornis crossleyi | A0A7L2SWB1 | A0A7L2SW96 | A0A7L2SSI2 | A0A7L2SVB0 |
| 198 | Naja naja | A0A8C6Y0L9 | A0A8C6Y0K8 | A0A8C6Y3I2 | A0A8C6Y1Q5 |
| 199 | Nannospalax galili | A0A8C6QRY3 | A0A8C6QEQ6 | A0A8C6R0V0 | A0A8C6RZS4 |
| 200 | Neodrepanis coruscans | A0A7L2RB74 | A0A7L2RB55 | A0A7L2RY02 | A0A7L2RUR8 |
| 201 | Neomonachus schauinslandi | A0A2Y9GQQ1 | A0A2Y9GQR4 | A0A2Y9GR00 | A0A2Y9GQW7 |
| 202 | Neophocaena asiaeorientalis asiaeorientalis | A0A341DDI6 | A0A341DD76 | A0A341DCP2 | A0A341DDI3 |
| 203 | Neopipo cinnamomea | A0A7K4QXM8 | A0A7K4QXA8 | A0A7K4QXM1 | A0A7K4QYJ1 |
| 204 | Nesospiza acunhae | A0A7K7REQ3 | A0A7K7RFR2 | A0A7K7RER1 | A0A7K7RGR3 |
| 205 | Nicator chloris | A0A852HGA7 | A0A852HKL5 | A0A852IAW1 | A0A852IBH4 |
| 206 | Notechis scutatus | A0A6J1TQK7 | A0A6J1TZ86 | A0A6J1TQG2 | A0A6J1TZ33 |
| 207 | Nothocercus julius | A0A7K7W7C1 | A0A7K7W558 | A0A7K7W7R3 | A0A7K7W7R6 |
| 208 | Nothocercus nigrocapillus | A0A851TFH9 | A0A851TFE2 | A0A851TDY0 | A0A851TF05 |
| 209 | Nothoprocta ornata | A0A7K7BBF6 | A0A7K7BBW8 | A0A7K7BBU4 | A0A7K7BBW1 |
| 210 | Notiomystis cincta | A0A7K6VMX5 | A0A7K6VMX0 | A0A7K6VP83 | A0A7K6VMZ4 |
| 211 | Nyctibius bracteatus | A0A7K8STH6 | A0A7K8SRY5 | A0A7K8SR04 | A0A7K8SSK2 |
| 212 | Nyctibius grandis | A0A7L2G9U1 | A0A7L2GB43 | A0A7L2GX45 | A0A7L2GX29 |
| 213 | Nycticryphes semicollaris | A0A7L1HVT3 | A0A7L1HXK0 | A0A7L1HYV4 | A0A7L1HW53 |
| 214 | Nyctiprogne leucopyga | A0A7L4C882 | A0A7L4C831 | A0A7L4CA23 | A0A7L4C8M2 |
| 215 | Oceanites oceanicus | A0A7K8UE26 | A0A7K8UDY0 | A0A7K8UFB7 | A0A7K8UDZ7 |
| 216 | Oceanodroma tethys | A0A7K9M9H1 | A0A7K9M8X9 | A0A7K9M8E9 | A0A7K9M9R3 |
| 217 | Octodon degus | A0A6P3FSS9 | A0A6P6DIE9 | A0A6P3FP92 | A0A6P3FDU9 |
| 218 | Odocoileus virginianus texanus | A0A6J0WX31 | A0A6J0WWY7 | A0A6J0X1N3 | A0A6J0WWX7 |
| 219 | Oenanthe oenanthe | A0A7L1E7D0 | A0A7L1E720 | A0A7L1E773 | A0A7L1E9E3 |
| 220 | Onychorhynchus coronatus | A0A7K5ZYS6 | A0A7K5ZYC8 | A0A7K5ZY86 | A0A7K5ZY44 |
| 221 | Oreocharis arfaki | A0A7K6JV21 | A0A7K6JW74 | A0A7K6JV41 | A0A7K6JV55 |
| 222 | Oreotrochilus melanogaster | A0A7L3N702 | A0A7L3N460 | A0A7L3N381 | A0A7L3N356 |
| 223 | Oriolus oriolus | A0A7L1P4D1 | A0A7L1P3B3 | A0A7L1P529 | A0A7L1P3B8 |
| 224 | Ornithorhynchus anatinus | F7DD13 | F7CTQ7 | F7DD20 | F7CS19 |
| 225 | Orthonyx spaldingii | A0A7K8G1N9 | A0A7K8G0V7 | A0A7K8G0W5 | A0A7K8G225 |
| 226 | Orycteropus afer afer | A0A8B6ZWK3 | A0A8B6ZRL7 | A0A8B6ZTA6 | A0A8B6ZRN8 |
| 227 | Otolemur garnettii | H0WR19 | H0XI31 | H0WLT5 | H0WLT6 |
| 228 | Oxylabes madagascariensis | A0A7L2R7U3 | A0A7L2R7F3 | A0A7L2R7T3 | A0A7L2RA82 |
| 229 | Oxyruncus cristatus | A0A7L1A702 | A0A7L1AG23 | A0A7L1AA63 | A0A7L1A901 |
| 230 | Pachycephala philippinensis | A0A7K9P8V8 | A0A7K9P9Z6 | A0A7K9P8V5 | A0A7K9P8L3 |
| 231 | Pachyramphus minor | A0A7K5DLK8 | A0A7K5DKJ8 | A0A7K5DJX1 | A0A7K5DK80 |
| 232 | Pandion haliaetus | A0A7L2WRD5 | A0A7L2WU64 | A0A7L2WVQ6 | A0A7L2WR96 |
| 233 | Panthera leo | A0A8C9D3G1 | A0A8C8X3J6 | A0A8C9D8X3 | A0A8C8XPD1 |
| 234 | Panthera pardus | A0A6P4VGG0 | A0A6P4VDD4 | A0A6P4VB46 | A0A6P4VG69 |
| 235 | Panthera tigris altaica | A0A8C9J1U1 | A0A8C9JJ34 | A0A8C9J1L6 | A0A8C9JRR6 |
| 236 | Panurus biarmicus | A0A7K6M2E0 | A0A7K6M316 | A0A7K6M261 | A0A7K6M239 |
| 237 | Papio anubis | A0A096MLT4 | A0A096MY47 | A0A096N640 | A0A096MKK8 |
| 238 | Passerina amoena | A0A852D2R1 | A0A852D3F0 | A0A852DC30 | A0A852DSG3 |
| 239 | Patagioenas fasciata monilis | A0A1V4KLV0 | A0A1V4KM00 | A0A1V4KM14 | A0A1V4KLW0 |
| 240 | Pedionomus torquatus | A0A7K6NX55 | A0A7K6NX20 | A0A7K6NX48 | A0A7K6NX04 |
| 241 | Pelecanoides urinatrix | A0A7L3BWX1 | A0A7L3BZX0 | A0A7L3BXF1 | A0A7L3BZS1 |
| 242 | Peromyscus maniculatus bairdii | A0A6I9MD93 | A0A6I9M1Q4 | A0A6I9M978 | A0A6I9MIH2 |
| 243 | Peucedramus taeniatus | A0A852G1V7 | A0A852FXB5 | A0A852FWA3 | A0A852FWU2 |
| 244 | Phaetusa simplex | A0A7L4AS98 | A0A7L4AQF1 | A0A7L4ARV9 | A0A7L4ATA1 |
| 245 | Phainopepla nitens | A0A7L1UEC3 | A0A7L1UB03 | A0A7L1U7U5 | A0A7L1U7T6 |
| 246 | Phascolarctos cinereus | A0A6P5JXK4 | A0A6P5JQT9 | A0A6P5JQS9 | A0A6P5JQG8 |
| 247 | Phocoena sinus | A0A8C9CCY0 | A0A8C9CDE7 | A0A8C9CCJ7 | A0A8C9CL66 |
| 248 | Phyllostomus discolor | A0A7E6D3E0 | A0A7E6D3J4 | A0A6J2KXT2 | A0A6J2L115 |
| 249 | Physeter macrocephalus | A0A2Y9FBX4 | A0A2Y9FC52 | A0A2Y9F421 | A0A2Y9FBV7 |
| 250 | Piaya cayana | A0A850X455 | A0A850XUM2 | A0A850WPX8 | A0A850WMZ5 |
| 251 | Picathartes gymnocephalus | A0A851AWP0 | A0A851AZN7 | A0A851AZT4 | A0A851B0A5 |
| 252 | Piliocolobus tephrosceles | A0A8C9I2H6 | A0A8C9I5Q3 | A0A8C9LUG4 | A0A8C9I3A9 |
| 253 | Piprites chloris | A0A7L0ISZ0 | A0A7L0IRU5 | A0A7L0IRX3 | A0A7L0IT47 |
| 254 | Pitta sordida | A0A851EXF9 | A0A851EXD9 | A0A851F0T6 | A0A851EVC1 |
| 255 | Platysteira castanea | A0A7K5V9L3 | A0A7K5V8A3 | A0A7K5VEN7 | A0A7K5V728 |
| 256 | Ploceus nigricollis | A0A7L0YQA1 | A0A7L0YTI0 | A0A7L0YTG9 | A0A7L0YQ90 |
| 257 | Pluvianellus socialis | A0A7L3DQ35 | A0A7L3DS05 | A0A7L3D8A9 | A0A7L3D7V0 |
| 258 | Podarcis muralis | A0A670IRE1 | A0A670IS45 | A0A670ITP2 | A0A670ITP8 |
| 259 | Podargus strigoides | A0A7L4GZU3 | A0A7L4H018 | A0A7L4H1W7 | A0A7L4H039 |
| 260 | Podilymbus podiceps | A0A7L0STD9 | A0A7L0SW96 | A0A7L0STC5 | A0A7L0STZ0 |
| 261 | Poecile atricapillus | A0A7K7R6L4 | A0A7K7R7B3 | A0A7K7R897 | A0A7K7R7S8 |
| 262 | Pogona vitticeps | A0A6J0SMH7 | A0A6J0SRN3 | A0A6J0SRM2 | A0A6J0SRM3 |
| 263 | Polioptila caerulea | A0A7K5EVK1 | A0A7K5EVJ1 | A0A7K5EWB3 | A0A7K5EVA6 |
| 264 | Pomatorhinus ruficollis | A0A7L4J984 | A0A7L4JBP3 | A0A7L4JAE2 | A0A7L4JCH9 |
| 265 | Pomatostomus ruficeps | A0A7L2SYQ8 | A0A7L2SXG3 | A0A7L2SZ71 | A0A7L2T0D3 |
| 266 | Pongo abelii | H2PBS1 | H2PBS0 | A0A2J8WT22 | H2PBR9 |
| 267 | Probosciger aterrimus | A0A7K5FSP1 | A0A7K5FT23 | A0A7K5FTI1 | A0A7K5FU92 |
| 268 | Prolemur simus | A0A8C8YUL0 | A0A8C8YLP0 | A0A8C8YLR0 | A0A8C8YLQ0 |
| 269 | Promerops cafer | A0A7K6XSH4 | A0A7K6XSF9 | A0A7K6X339 | A0A7K6X3B9 |
| 270 | Prunella himalayana | A0A7K5QSD9 | A0A7K5QS00 | A0A7K5QRZ9 | A0A7K5QSJ2 |
| 271 | Pseudonaja textilis | A0A670YDP3 | A0A670Y3P5 | A0A670Y3P1 | A0A670YB63 |
| 272 | Psophia crepitans | A0A7K9XUG2 | A0A7K9XTP7 | A0A7K9XWQ6 | A0A7K9XVE2 |
| 273 | Pterocles burchelli | A0A7K5YJ56 | A0A7K5YJK3 | A0A7K5YJ04 | A0A7K5YJS2 |
| 274 | Pteropus vampyrus | A0A6P3QZI3 | A0A6P6BKY8 | A0A6P6BKY7 | A0A6P3R0Y3 |
| 275 | Pteruthius melanotis | A0A852NAW4 | A0A852MVR5 | A0A852N8F9 | A0A852N4Z8 |
| 276 | Ptilonorhynchus violaceus | A0A7K6C2R7 | A0A7K6C569 | A0A7K6C4H1 | A0A7K6C5H5 |
| 277 | Ptilorrhoa leucosticta | A0A7K8M7X7 | A0A7K8M796 | A0A7K8M7I0 | A0A7K8M7Y0 |
| 278 | Puma concolor | A0A6P6H0M3 | A0A6P6GZV1 | A0A6P6H053 | A0A6P6GZW2 |
| 279 | Pycnonotus jocosus | A0A7L2P0J8 | A0A7L2P0C4 | A0A7L2P086 | A0A7L2P2Q6 |
| 280 | Quiscalus mexicanus | A0A7L2F1W9 | A0A7L2EU12 | A0A7L2EXD5 | A0A7L2EVB7 |
| 281 | Ramphastos sulfuratus | A0A852C466 | A0A852C2N5 | A0A852BYP2 | A0A852C2J7 |
| 282 | Rattus norvegicus | Q9EPX4 | Q6GUG4 | O35881 | F1LXU3 |
| 283 | Regulus satrapa | A0A7K4XX00 | A0A7K4XX42 | A0A7K4XX34 | A0A7K4XX06 |
| 284 | Rhabdornis inornatus | A0A7K9KU27 | A0A7K9KT81 | A0A7K9KVY1 | A0A7K9KWJ5 |
| 285 | Rhadina sibilatrix | A0A7L2NE48 | A0A7L2NDY7 | A0A7L2NDN5 | A0A7L2NE09 |
| 286 | Rhagologus leucostigma | A0A7K8BQ18 | A0A7K8BNB0 | A0A7K8C4E4 | A0A7K8BQI2 |
| 287 | Rhinolophus ferrumequinum | A0A671EKS0 | A0A671EKM9 | A0A671EL85 | A0A671ELA8 |
| 288 | Rhinopithecus bieti | A0A2K6KZ31 | A0A2K6K1J2 | A0A2K6LK93 | A0A2K6M7L2 |
| 289 | Rhinopithecus roxellana | A0A2K6RG34 | A0A2K6RBN3 | A0A2K6NKH8 | A0A2K6PT52 |
| 290 | Rhinopomastus cyanomelas | A0A7L1NYS3 | A0A7L1NYR9 | A0A7L1P0E0 | A0A7L1P1Y8 |
| 291 | Rhipidura dahli | A0A7K9WLE8 | A0A7K9WLS2 | A0A7K9WM23 | A0A7K9WLC0 |
| 292 | Rhodinocichla rosea | A0A7K8RYP2 | A0A7K8RYP0 | A0A7K8S9U2 | A0A7K8S9M3 |
| 293 | Rhynochetos jubatus | A0A7K6RXA1 | A0A7K6RXC4 | A0A7K6RXB2 | A0A7K6RXL5 |
| 294 | Rissa tridactyla | A0A7L3SN25 | A0A7L3SQX5 | A0A7L3SMB9 | A0A7L3SNF8 |
| 295 | Rostratula benghalensis | A0A7L0CU51 | A0A7L0CV28 | A0A7L0CUA7 | A0A7L0CX11 |
| 296 | Rynchops niger | A0A7L1KG82 | A0A7L1KFZ9 | A0A7L1KG23 | A0A7L1KFR7 |
| 297 | Sagittarius serpentarius | A0A7L2HNC3 | A0A7L2HQA9 | A0A7L2HNC6 | A0A7L2HRI6 |
| 298 | Sakesphorus luctuosus | A0A7K8Z6G5 | A0A7K8Z4A5 | A0A7K8Z6F5 | A0A7K8Z5B0 |
| 299 | Salvator merianae | A0A8D0BCV1 | A0A8D0BC47 | A0A8D0BDW5 | A0A8D0BBL4 |
| 300 | Sapajus apella | A0A6J3GM98 | A0A6J3GLU3 | A0A6J3GMG0 | A0A6J3GLS8 |
| 301 | Sapayoa aenigma | A0A7K7SR08 | A0A7K7SRS1 | A0A7K7STV1 | A0A7K7SQY6 |
| 302 | Sarcophilus harrisii | A0A7N4NG46 | A0A7N4P8E1 | G3WZZ8 | G3WZK8 |
| 303 | Sciurus vulgaris | A0A8D2B127 | A0A8D2B384 | A0A8D2B189 | A0A8D2CP75 |
| 304 | Sclerurus mexicanus | A0A7K8WEB3 | A0A7K8WEH4 | A0A7K8WEC2 | A0A7K8WEK2 |
| 305 | Scopus umbretta | A0A7L4IFA8 | A0A7L4IDX2 | A0A7L4HJP8 | A0A7L4HV84 |
| 306 | Scytalopus superciliaris | A0A7L1YEM3 | A0A7L1YHG8 | A0A7L1YDF9 | A0A7L1YDD5 |
| 307 | Semnornis frantzii | A0A7L2IGJ3 | A0A7L2IED7 | A0A7L2IGR4 | A0A7L2IRK5 |
| 308 | Serilophus lunatus | A0A7L1CR13 | A0A7L1CPC7 | A0A7L1CVC9 | A0A7L1CXF8 |
| 309 | Serinus canaria | A0A8C9UD04 | A0A8C9N2G4 | A0A8C9UCG1 | A0A8C9N0V1 |
| 310 | Setophaga kirtlandii | A0A7L0R8Q6 | A0A7L0R760 | A0A7L0QFB6 | A0A7L0R9B2 |
| 311 | Sitta europaea | A0A7L1VA94 | A0A7L1VAV9 | A0A7L1VBS3 | A0A7L1VBR0 |
| 312 | Smithornis capensis | A0A7K8QBH2 | A0A7K8QDH9 | A0A7K8QBI9 | A0A7K8QCS2 |
| 313 | Smutsornis africanus | A0A7L1J3X2 | A0A7L1J3B7 | A0A7L1J3J7 | A0A7L1J3B0 |
| 314 | Spheniscus mendiculus | A0A8J4MJZ2 | A0A8J4I425 | A0A8J4I2Y3 | A0A8J4I306 |
| 315 | Spizaetus tyrannus | A0A7L0BNC2 | A0A7L0BNC7 | A0A7L0BQ43 | A0A7L0BNH2 |
| 316 | Spizella passerina | A0A852JSB3 | A0A852K304 | A0A852K049 | A0A852K7P3 |
| 317 | Steatornis caripensis | A0A7K6WL84 | A0A7K6WK51 | A0A7K6WN45 | A0A7K6WJS2 |
| 318 | Stercorarius parasiticus | A0A7K9FJ93 | A0A7K9FJI2 | A0A7K9FJN2 | A0A7K9FL21 |
| 319 | Sterrhoptilus dennistouni | A0A7K9S704 | A0A7K9S626 | A0A7K9S7X7 | A0A7K9S614 |
| 320 | Strigops habroptila | A0A672VC50 | A0A672VEZ2 | A0A672VBH6 | A0A672VC70 |
| 321 | Struthidea cinerea | A0A7K8FCN0 | A0A7K8FCL2 | A0A7K8FDX3 | A0A7K8FDL7 |
| 322 | Sula dactylatra | A0A850ZZ99 | A0A850ZWB7 | A0A850ZXB1 | A0A850ZVA4 |
| 323 | Sus scrofa | D3Y271 | A0A287A083 | F1SJN3 | A0A287AN17 |
| 324 | Sylvia atricapilla | A0A7K7DSQ8 | A0A7K7DUS8 | A0A7K7DSN1 | A0A7K7DSP8 |
| 325 | Sylvietta virens | A0A7L0LGS7 | A0A7L0LEB7 | A0A7L0LET0 | A0A7L0LG32 |
| 326 | Syrrhaptes paradoxus | A0A7L3AHC0 | A0A7L3AFS5 | A0A7L3AC81 | A0A7L3ADU7 |
| 327 | Tachuris rubrigastra | A0A7K4WM96 | A0A7K4WMA1 | A0A7K4WMC7 | A0A7K4WM62 |
| 328 | Taeniopygia guttata | A0A674HH83 | A0A674GUN7 | A0A674GAZ5 | H1A4B5 |
| 329 | Terrapene carolina triunguis | A0A674I7H3 | A0A674I356 | A0A674I352 | A0A674I500 |
| 330 | Thalassarche chlororhynchos | A0A7L3J315 | A0A7L3J3M0 | A0A7L3J5H2 | A0A7L3J1J8 |
| 331 | Theropithecus gelada | A0A8D2ENN6 | A0A8D2EKN8 | A0A8D2EJS7 | A0A8D2JWW0 |
| 332 | Thinocorus orbignyianus | A0A7L1XV91 | A0A7L1XVS9 | A0A7L1XVT0 | A0A7L1XRQ6 |
| 333 | Thryothorus ludovicianus | A0A7K7YZX0 | A0A7K7YXK3 | A0A7K7YXD2 | A0A7K7YWM6 |
| 334 | Tichodroma muraria | A0A850YWI7 | A0A850YZ71 | A0A850YVX1 | A0A850YVY0 |
| 335 | Todus mexicanus | A0A851DWY2 | A0A851D3T2 | A0A851DVC2 | A0A851DNB6 |
| 336 | Toxostoma redivivum | A0A7K5IPG0 | A0A7K5IP70 | A0A7K5IP81 | A0A7K5IPK2 |
| 337 | Tricholaema leucomelas | A0A852IMA5 | A0A852IIU2 | A0A852J4L5 | A0A852IMY0 |
| 338 | Trogon melanurus | A0A7L0EB42 | A0A7L0EAQ2 | A0A7L0EBE2 | A0A7L0EB36 |
| 339 | Turnix velox | A0A7L3LBJ8 | A0A7L3LAQ4 | A0A7L3LAU1 | A0A7L3LCT4 |
| 340 | Tursiops truncatus | A0A2U4B9N1 | A0A2U4BA25 | A0A2U3V8S4 | A0A2U3V9B4 |
| 341 | Tychaedon coryphoeus | A0A851R8G7 | A0A851R5Q2 | A0A851R1W9 | A0A851R9M9 |
| 342 | Tyrannus savana | A0A7L0X2I7 | A0A7L0X4M3 | A0A7L0X3K8 | A0A7L0X3Q5 |
| 343 | Upupa epops | A0A7K6B1X3 | A0A7K6B214 | A0A7K6B1Z3 | A0A7K6B384 |
| 344 | Urocitellus parryii | A0A8D2KIF8 | A0A8D2HII3 | A0A8D2HIH3 | A0A8D2HTC0 |
| 345 | Urocolius indicus | A0A852L797 | A0A852L027 | A0A852L475 | A0A852L9W0 |
| 346 | Urocynchramus pylzowi | A0A7K5SSV5 | A0A7K5SRC1 | A0A7K5T4D3 | A0A7K5SSZ0 |
| 347 | Ursus americanus | A0A452R191 | A0A452R1B3 | A0A452RT99 | A0A452R170 |
| 348 | Ursus maritimus | A0A384DEY0 | A0A384DEW0 | A0A384DE86 | A0A452U749 |
| 349 | Vicugna pacos | A0A6J0AH84 | A0A6I9I6J5 | A0A6J3ANL2 | A0A6I9I3W1 |
| 350 | Vidua chalybeata | A0A851KJF3 | A0A851KK79 | A0A851KKZ4 | A0A851KHH3 |
| 351 | Vidua macroura | A0A852EC36 | A0A852EB70 | A0A852EC45 | A0A852EGH2 |
| 352 | Vireo altiloquus | A0A7K5KSL9 | A0A7K5KSI8 | A0A7K5KSF8 | A0A7K5KU91 |
| 353 | Vombatus ursinus | A0A4X2KTA5 | A0A4X2L186 | A0A4X2L169 | A0A4X2L175 |
| 354 | Vulpes vulpes | A0A3Q7UEB7 | A0A3Q7UVV8 | A0A3Q7UVV5 | A0A3Q7U1K3 |
| 355 | Xenopus laevis | Q5XG06 | Q6DCY2 | A0A1L8G4A8 | A0A1L8G4G6 |
| 356 | Xenopus tropicalis | F7CTF7 | Q5FWR3 | A0A803K3W5 | A0A803J4D2 |
| 357 | Xiphorhynchus elegans | A0A7L3PJK2 | A0A7L3PJJ9 | A0A7L3PPP4 | A0A7L3PJJ6 |
| 358 | Zalophus californianus | A0A6J2CCL9 | A0A6J2C6V5 | A0A6J2CCL6 | A0A6J2C8S5 |
| 359 | Zapornia atra | A0A7L3F4Z8 | A0A7L3F049 | A0A7L3F0D7 | A0A7L3F213 |
